# Prevalence and Risk Factors of Heart Failure in Patients Diagnosed with Hyperthyroidism: A Systematic Review and Meta-analysis

**DOI:** 10.17925/EE.2024.20.2.12

**Published:** 2024-07-22

**Authors:** Si Wei David Fan, Leong Tung Ong

**Affiliations:** Faculty of Medicine, University of Malaya, Kuala Lumpur, Malaysia

**Keywords:** Graves’ disease, heart failure, hyperthyroidism, prevalence, risk factors, treatment

## Abstract

**Objectives:** Hyperthyroidism has a significant impact on the cardiovascular system, causing thyrotoxic cardiomyopathy, which is characterized by atrial fibrillation, left ventricular hypertrophy and diastolic dysfunction, and may lead to heart failure. This study aimed to investigate the prevalence and associated risk factors for heart failure in patients with hyperthyroidism. **Methods:** A systematic literature search was conducted on PubMed, SCOPUS and Ovid SP up until April 2023. Pooled prevalence and pooled odds ratio for risk factors were calculated using the generic inverse variance method. **Results:** Studies involving 30,889 patients were included in this meta-analysis. The overall prevalence of heart failure in patients with hyperthyroidism was 8% (95% confidence interval [CI]: 6–11%). Further analyses revealed that the prevalence of heart failure in patients who underwent treatment with radioactive iodine ablation, antithyroid medication and thyroidectomy was 8% (95% CI: -1 to 16%), 6% (95% CI: 2 to 11%) and 4% (95% CI: -2 to 10%), respectively. The risk factors of heart failure in hyperthyroidism include atrial fibrillation, chronic kidney disease, anaemia, hypertension, history of stroke or transient ischaemic attack, history of coronary artery disease and diabetes mellitus. **Conclusion:** Heart failure occurs in 8% of patients with hyperthyroidism, with the most common risk factor being atrial fibrillation.

Hyperthyroidism is prevalent in 0.1–2.5% of the population, and Graves’ disease is diagnosed in 80% of patients with hyperthyroidism.^1,2^ This condition arises from the uncontrolled, excessive activation of the thyroid-stimulating hormone (TSH) receptor by autoreactive TSH-receptor antibodies.^2^ Elevated thyroid hormones in hyperthyroidism result in a hyperdynamic circulatory state, leading to an increase in cardiac contractility, resting heart rate and cardiac output while decreasing the peripheral vascular resistance and isovolumic relaxation time.^3^ Patients with uncontrolled and prolonged hyperthyroidism may exacerbate pre-existing cardiac disease or directly cause thyrotoxic cardiomyopathy, leading to heart failure, atrial fibrillations and pulmonary hypertension.^4–7^

The cardiovascular complications in Graves’ disease may lead to a 20% increased mortality rate in overt hyperthyroidism, and the mortality rate is as high as 50% in cases of severe thyrotoxicosis.^8,9^ Approximately 50% of patients with hyperthyroidism diagnosed with congestive heart failure had impaired left ventricular systolic function, which is similar to congestive heart failure in the general population.^10^ In addition, the development of congestive heart failure in overt hyperthyroidism may be attributed to left ventricular diastolic dysfunction, which has been shown to be a predictor of heart failure.^11^ Patients who develop heart failure as a result of hyperthyroidism have a favourable prognosis and outcome, often achieving complete recovery of heart function; this is in contrast to the majority of the population, where heart failure frequently occurs due to ischaemic heart disease.^12^ The resolution of heart failure in patients with Graves’ disease has been observed upon attaining a euthyroid state, indicating the potential reversibility with appropriate hyperthyroidism treatment.^13,14^

The available treatment approaches for hyperthyroidism include antithyroid medication, radioactive iodine ablation and thyroidectomy. Among these, antithyroid medication is the most common first-line treatment.^15^ There is a significant reduction in the incidence of cardiac diseases in patients with Graves’ disease after antithyroid medication treatment, with 71% experiencing the resolution of congestive heart failure.^16^ However, the significant limitation of antithyroid medication is its low remission rate of approximately 30–40% within a duration of 12–18 months, posing a challenge given the crucial need for the prompt resolution of hyperthyroidism in patients with heart failure.^15,17^ Therefore, thyroidectomy may be the more appropriate first-l ine treatment in patients who have developed cardiovascular complications as the patients can achieve a euthyroid state in a shorter duration of time and lower rates of relapse compared with antithyroid medication and radioactive iodine ablation. However, there are limited data available on the incidence, clinical characteristics and risk factors for heart failure associated with hyperthyroidism. The aim of this study is to investigate the prevalence and risk factors of heart failure among patients diagnosed with hyperthyroidism.

## Methods

### Search strategies

A systematic literature search was conducted on PubMed, SCOPUS and Ovid SP through April 2023 using the following search strategy: (hyperthyroidism OR Graves’ OR goitre OR goiter) AND (heart failure OR heart decompensation OR ventricular dysfunction OR diastolic dysfunction OR systolic dysfunction). Furthermore, the authors also retrieved additional articles from reference lists and selected the relevant studies to optimize the search. The search strategy was limited to studies published in the English language and peer-reviewed journals.

### Eligibility criteria

The criteria for inclusion for this meta-analysis include the following: (i) observational cohort studies (prospective or retrospective), case– control studies and cross-sectional studies; (ii) studies involving adult patients; (iii) diagnosis of hyperthyroidism; (iv) diagnosis of heart failure and (v) reporting the prevalence data of heart failure in patients with hyperthyroidism. The criteria for exclusion for this meta-analysis include the following: (i) reviews, systematic reviews, meta-analysis, editorials, case reports or case series; (ii) patients with cardiomyopathies and (iii) studies that did not report the relevant data or outcomes on the prevalence of heart failure. The search strategy was limited to studies published in the English language and in peer-reviewed journals.

### Study selection and data extraction

The authors independently screened all the titles and abstracts of the articles based on the eligibility criteria to be included in this meta-analysis. The full text of the article was used for review when the eligibility for inclusion based on titles and abstracts was inconclusive. The full text of all the relevant articles was then reviewed against the inclusion criteria to be included in the meta-analysis. Any discrepancies or disagreements regarding the eligibility criteria were resolved through discussion and consensus between both authors. The authors extracted the relevant data of the included studies independently into a pre-designed standardized electronic form created in Microsoft Excel. The extracted data from the studies included the following: the first author’s name; year; country; study design; age; gender; total sample size; diagnostic criteria for heart failure; prevalence data for heart failure; sample size and prevalence data for each treatment groups of antithyroid medication, radioactive iodine ablation and thyroidectomy and baseline free T4 (FT4) level, baseline total/free T3 level and TSH level.

### Quality assessment

The methodological quality and the risk of bias of the included studies were evaluated based on the Newcastle–Ottawa Scale.^18^ The quality of the studies was assessed based on the study selection (representativeness of the exposed cohort, selection of the non-exposed cohort, ascertainment of exposure and demonstration that the outcome of interest was not present at the start of the study), comparability (comparability of cohorts on the basis of the design and analysis) and outcome (assessment of outcome, was followed up long enough for outcomes to occur and adequacy of follow-up of cohorts). Each criterion received a score of 1, with a maximum possible score of 2 for comparability, resulting in a total potential score of 9. The Newcastle– Ottawa scores for each study were then converted into the Agency for Healthcare Research and Quality standards of good, fair and poor. The qualities of each of the original articles were evaluated independently by both authors, and discrepancies were resolved through discussion.

### Data analysis

The primary endpoint of this study was the prevalence of heart failure in patients with hyperthyroidism. The secondary endpoints of this study were the prevalence of heart failure in each treatment group of antithyroid medications, surgery and radioactive iodine ablation and the predictors of heart failure. The prevalence of heart failure was expressed as the percentage. The pooled prevalence with a 95% confidence interval (CI) was calculated using the random-effects generic inverse variance model.^19^ The standard error (SE) for prevalence was calculated using the following formula: SE=√*p* (1-*p*)/n, 95% CI=*p* ± 1.96 x SE, where p=prevalence and n=sample size. Heterogeneity between studies was determined using the chi-squared test, with the degree of heterogeneity quantified by *I*^2^.^20^ *I*^2^ values of >25%, >50% and >75% correspond to a low, moderate and high degree of heterogeneity effects, respectively.^20^ For the risk factors of heart failure, the odds ratio (OR) of each variable was calculated. The pooled OR for each variable was calculated using the generic inverse variance model. For publication bias, visual inspection of the funnel plot and Egger’s regression test were used.^21^ A p-value of <0.05 was considered statistically significant. Statistical analysis was performed using the Cochrane Review Manager v5.4 and R programming with the *metafor* package.^22–24^ The findings of this meta-analysis were reported according to the Preferred Reporting Items for Systematic Reviews and Meta-Analyses (PRISMA) guidelines.^25^

## Results

### Search results and eligible studies

**Figure 1:**
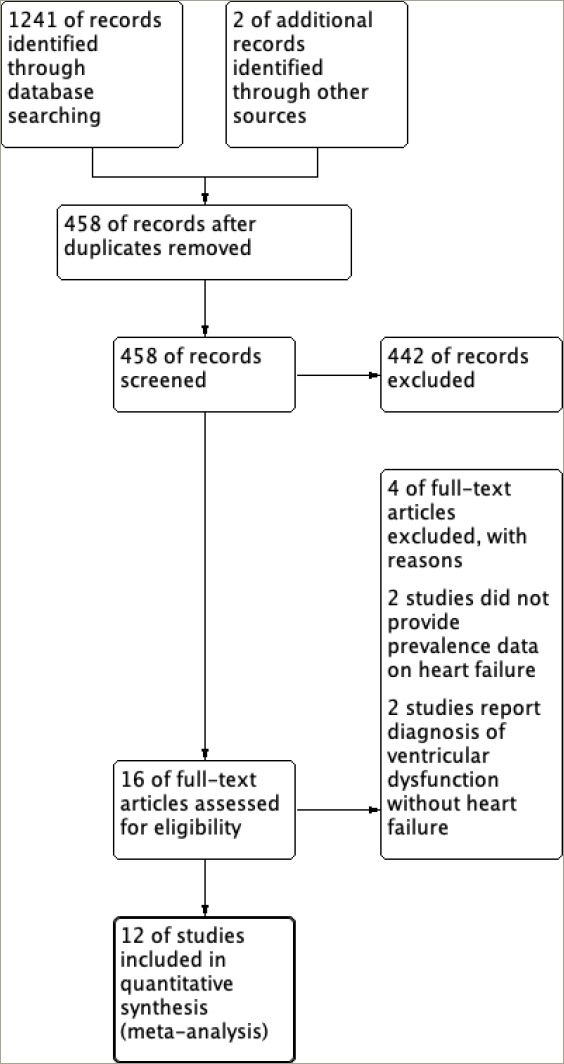
Preferred Reporting Items for Systematic Reviews and Meta-Analyses flow diagram

The search strategy identified a total of 1,241 articles, with an additional 2 articles identified through the bibliographies of the selected review articles. A total of 458 articles were screened based on their titles and abstracts following the removal of duplicate entries. A full-text review was then carried out on 16 studies to determine their eligibility. Four were excluded out of the 16 studies: 2 did not provide the data regarding the prevalence of heart failure and 2 reported the prevalence of ventricular dysfunction without clinical diagnosis of heart failure. Consequently, 12 eligible studies were included in this meta-analysis. A detailed flow chart illustrating the study selection process following the PRISMA flow diagram is depicted in *Figure 1*.

### Study characteristics

Twelve studies consisting of 30,889 patients diagnosed with hyperthyroidism were included in this meta-analysis. The sample size of the included studies ranged from 24 to 16,882 patients.^10,12,15,16,26–33^ For the study design, three were case–control studies, three were prospective cohort studies, five were retrospective cohort studies and one was a cross-sectional study. Six studies reported the mean age, and six studies reported the median age, which ranged from 37.9 to 62.0 years. All the studies reported that the majority of participants were female, and the percentage of male participants ranged from 9 to 34%. Five of the included studies utilized the diagnostic criteria for heart failure, where three studies used modified Framingham criteria, one study used European Society of Cardiology (ESC) guidelines, and one study used clinical diagnosis.^34,35^ Four studies were conducted in Asian countries, European countries and North American countries. An overview of the characteristics of the included studies is summarized in *Table 1*.^10,12,15,16,26–33^

### Critical appraisal of studies

The study quality according to the Newcastle–Ottawa Scale is shown in *Table 2*.^10,12,15,16,26–33^ The scores of the studies ranged from 5 to 9. Nine studies were of good quality and three studies were of poor quality. Three studies were rated as poor quality because they did not conduct follow-ups on the patients. Consequently, the assessment of follow-up adequacy and the occurrence of outcomes could not be conducted. The average score of the studies included was 7.5±1.09.

**Table 1:** Characteristics of the included studies^10,12,15,16,26–33^

Study	Country	Study design	Age (years)	Gender, male (percentage)	Diagnostic criteria for HF	Sample size	Prevalence of HF (number of participants)	Baseline free T4 level	Baseline free/total T3 level	Baseline TSH level (mIU/L)
Kage et al. (1993)^26^	Japan	Case–control study	49.0±16.2	2 (8)	Presence of an increased cardiothoracic ratio on chest radiogram, pretibial pitting oedema and dyspnoea	24	8	Not reported	Not reported	Not reported
Siu et al. (2007)^10^	Hong Kong	Prospective cohort study	45±1	140 (24)	Modified Framingham criteria	591	34	Not reported	Not reported	Not reported
Metso et al. (2008)^27^	Finland	Case–control study	62 (49–72)	Not reported	Not reported	2,611	346	Not reported	Not reported	Not reported
Ryödi et al. (2018)^28^	Finland	Case–control study	46 (33–59)	615 (14)	Not reported	4,334	169	Not reported	Not reported	Not reported
Tsymbaliuk et al. (2015)^16^	Ukraine	Prospective cohort study	52 (30–69)	15 (25)	ESC guidelines for the diagnosis and treatment of acute and chronic heart failure, 2012	61	12	81.3 (25.2–335) pmol/L	1.42 (0.54 – 2.48) pmol/L	0.023 (0.003–0.07) mIU/L
Gauthier et al. (2016)^12^	USA	Prospective cohort study	GD: 37.9±14.0 MNG: 39.8±11.3	GD: 5 (13) MNG: 5 (13)	Not reported	80	4	GD: 2.61 ng/dL MNG: 1.07 ng/dL	Not reported	GD: 0.56 U/mL MNG: 1.00 U/mL
Okosieme et al. (2019)^29^	UK	Retrospective cohort study	48±16	775 (19)	Not reported	4,189	109	31.8±22.0 pmol/L	Not reported	Not reported
Elnahla et al. (2021)^30^	USA	Retrospective cohort study	Surgical: 42.93±15.25 Medical: 42.52±14.94	Surgical: 8 (12) Medical: 24 (29)	Not reported	151	Surgical: 5 Medical: 5	Surgical: 2.82±2.5 ng/dL Medical: 3.86±5.15 ng/dL	Not reported	Surgical: 0.53±1.6 U/mL Medical: 0.05±0.18 U/mL
Song et al. (2021)^15^	South Korea	Retrospective cohort study	51 (42–60)	5,755 (34)	Not reported	16,882	AT: 1,379 RIA: 26	Not reported	Not reported	Not reported
Naser et al. (2022)^31^	USA	Retrospective cohort study	53 (38–66)	121 (24)	Modified Framingham criteria	495	HFrEF: 12	4.1 (2.1–9.3) ng/dL	Not reported	Not reported
Naser et al. (2022)^32^	USA	Retrospective cohort study	48 (34–62)	299 (22)	Modified Framingham criteria with HFrEF, LVEF <50%, and HFpEF, LVFF ≥50%	1,317	HFrEF: 31 HFpEF: 43	HFrEF: 3.6 (2.4–5.7) ng/dL HFpEF: 3.1 (2.2–4.7) ng/dL	HFrEF: 403 (261–543) pg/mL HFpEF: 238 (164–336) pg/mL	Not reported
Nijith et al. (2022)^33^	India	Clinical descriptive cross-sectional study	43.2±13.7	21 (15)	Not reported	140	20	Not reported	Not reported	Not reported

*AT = Antithyroid medication; ESC = European Society of Cardiology; GD = Graves’ disease; HF = heart failure; HFpEF = heart failure with preserved ejection fraction; HFrEF = heart failure with reduced ejection fraction; LVEF = left ventricular ejection fraction; MNG = multinodular goitre; RIA = Radioactive iodine ablation; TSH = thyroid-stimulating hormone.*

### Prevalence of heart failure in patients diagnosed with hyperthyroidism

**Figure 2:**
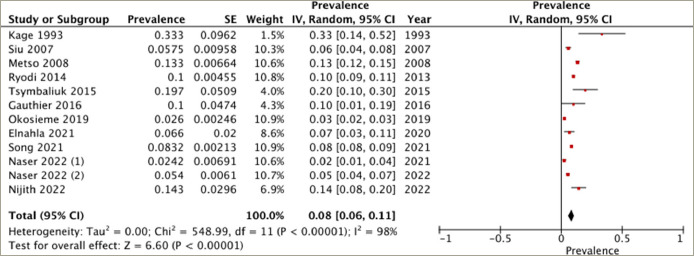
Figure 2: Prevalence of heart failure in patients diagnosed with hyperthyroidism

All 12 studies reported on the prevalence of heart failure in patients diagnosed with hyperthyroidism. The reported prevalence of heart failure in the studies exhibits substantial variation, ranging from 2.4 to 33.3%. The overall pooled prevalence of heart failure was 8% (95% CI: 6–11%). There was high heterogeneity between the studies (*I*^2^=98%, p<0.001) (*Figure 2*).

## Prevalence of heart failure in different treatment groups

**Figure 3:**
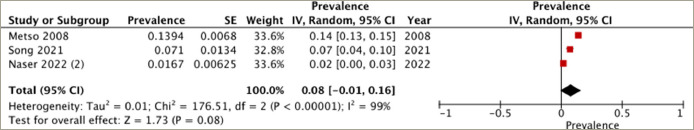
Prevalence of heart failure in the radioactive iodine ablation group

**Figure 4:**
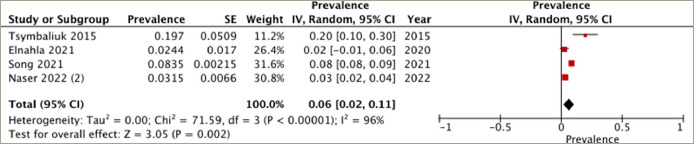
Prevalence of heart failure in the antithyroid medication group

**Figure 5:**
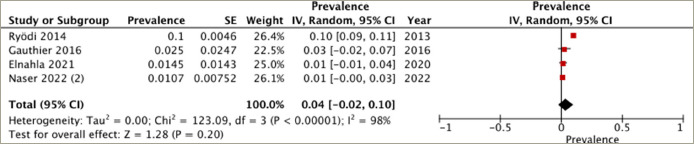
Prevalence of heart failure in the thyroidectomy group

A subgroup analysis was conducted on patients who underwent different treatment modalities, including antithyroid medication, thyroidectomy and radioactive iodine ablation. Four studies reported the prevalence of heart failure in each of the antithyroid medication and thyroidectomy groups, while three studies provided the data for the radioactive iodine ablation group. The highest prevalence of heart failure was observed in the radioactive iodine ablation group, with a pooled prevalence of 8% (95% CI: -1–16%), and substantial heterogeneity (*I*^2^=99%, p<0.001) (*Figure 3*). Following this, the antithyroid medication group demonstrated a pooled prevalence of 6% (95% CI: 2–11%) and substantial heterogeneity (*I*^2^=96%, p<0.001) (*Figure 4*). The thyroidectomy group demonstrated the lowest prevalence of heart failure at 4% (95% CI: -2–10%) and substantial heterogeneity (*I*^2^=98%, p<0.001) (*Figure 5*).

### Risk factors of heart failure in patients diagnosed with hyperthyroidism

Five studies compared the baseline characteristics of patients diagnosed with and without heart failure. The risk factors of developing heart failure in patients diagnosed with hyperthyroidism are summarized in *Table 3*. Patients with underlying medical comorbidities had higher odds of developing heart failure. Medical comorbidities, including atrial fibrillation (OR: 17.09, 95% CI: 4.86–60.17), chronic kidney disease (OR: 6.40, 95% CI: 3.72–11.01), anaemia (OR: 5.04, 95% CI: 3.17–8.00), hypertension (OR: 4.84, 95% CI: 2.72–8.62), history of stroke or transient ischaemic attack (OR: 4.28, 95% CI: 1.72–10.65), history of coronary artery disease (OR: 2.87, 95% CI: 1.37–5.99) and diabetes mellitus (OR: 2.36, 95% CI: 1.41–3.96), had a statistically significant increase in odds of developing heart failure. In contrast, gender and diagnosis of hyperthyroidism did not demonstrate an increased odds of developing heart failure. Risk factors, such as chronic obstructive pulmonary disease (OR: 4.15, 95% CI: 0.80–21.66), male gender (OR: 1.92, 95% CI: 0.63–5.83), diagnosis of goitre (OR: 1.89, 95% CI: 0.96–3.71), dyslipidaemia (OR: 1.48, 95% CI: 0.31–7.20), smoking (OR: 1.47, 95% CI: 0.65–3.32) and diagnosis of Graves’ disease (OR: 0.55, 95% CI: 0.65–3.32), did not have a significant increase in odds of developing heart failure.

**Table 2:** Newcastle–Ottawa Scale scores for the quality assessment of the included studies^10,12,15,16,26–33^

	Selection				Comparability	Outcome			
Study	Representativeness of the exposed cohort	Selection of the non-exposed cohort	Ascertainment of exposure	Demonstration that the outcome of interest was not present at the start of the study	Comparability cohorts on the basis of the design or analysis	Assessment of the outcome	Was follow-up enough for outcomes to occur	Adequacy of follow-up	Total (out of9)
Kage et al. (1993)^26^	*	*	*	*	**	*			7
Siu et al. (2007)^10^	*	*	*	*	**	*	*	*	9
Metso et al. (2008)^27^	*	*	*	*	*	*	*	*	8
Ryödi et al. (2018)^28^	*	*	*	*	*	*	*	*	8
Tsymbaliuk et al. (2015)^16^	*		*	*	*	*	*	*	7
Gauthier et al. (2016)^12^	*		*	*	*	*	*	*	7
Okosieme et al. (2019)^29^	*	*	*	*	*	*	*	*	8
Elnahla et al. (2021)^30^	*		*	*	*	*	*	*	7
Song et al. (2021)^15^	*	*	*	*	*	*	*	*	8
Naser et al. (2022)^31^	*	*	*	*	**	*	*	*	9
Naser et al. (2022)^32^	*	*	*	*	**	*			7
Nijith et al. (2022)^33^	*		*	*	*	*			5

* one point given

** two points given

### Publication bias

**Figure 6:**
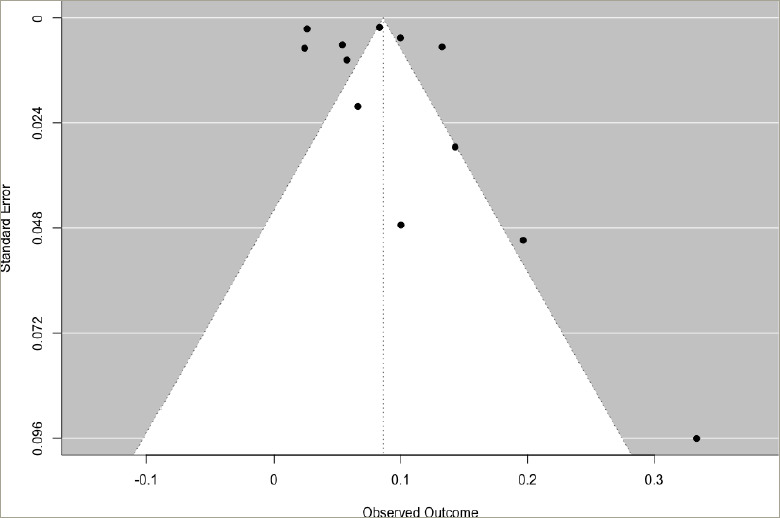
Funnel plot for prevalence of heart failure in hyperthyroidism

The prevalence of heart failure was examined for publication bias. The visual assessment of the funnel plot showed an asymmetrical distribution, indicating the presence of publication bias (*Figure 6*). Furthermore, Egger’s regression test showed that there was a potential risk of publication bias (p=0.016).

*CI = confidence interval; df = degrees of freedom; IV = inverse variance; SE = standard error.*

*CI = confidence interval; df = degrees of freedom; IV = inverse variance; SE = standard error.*

## Discussion

This meta-analysis found that the prevalence of heart failure is low in patients diagnosed with hyperthyroidism. The overall prevalence of heart failure was 8%. Furthermore, a subgroup analysis suggested that the radioactive iodine ablation group had the highest prevalence of heart failure compared with other treatment groups. Several medical comorbidities were found to increase the odds of developing heart failure in patients diagnosed with hyperthyroidism.

Thyroid hormones upregulate the alpha chain, downregulate the beta chain and directly influence ion channels, such as voltage-gated K^+^ channels, Na/K-ATPase and Na/Ca^2+^ exchanger, thereby impacting myocardial and vascular properties.^3,33^ Therefore, excess thyroid hormones may induce haemodynamic instability by enhancing cardiac output through increased contractility of cardiac muscles, elevated heart rate and decreased peripheral vascular resistance.^36^ Additionally, they can also act on the ion channels of cardiac pacemaker cells, thereby precipitating arrhythmias.^37^ Cardiovascular injury in Graves’ disease is characterized by antibody-mediated damage to the myocardium, resulting in functional hypertrophy attributed to TSH-receptor antibody.^3,36^ In addition, in patients with underlying Graves’ disease, autoimmune myocarditis has been proposed as a contributing factor to heart failure.^38,39^ Radioactive cardiac imaging and cardiovascular magnetic resonance imaging have demonstrated that hyperthyroidism can result in direct damage to the myocardium.^10^

This study demonstrated that heart failure occurred in 8% of the patients with hyperthyroidism and the prevalence was higher compared with that of the general population (1.5–1.6%).^40^ Excess thyroid hormone may exert toxic effects on the myocardial muscles, leading to left ventricular diastolic dysfunction, which is an independent predictor of developing heart failure.^11,41^ However, only 50% of patients with hyperthyroidism diagnosed with heart failure had reduced left ventricular ejection fraction.^11^ Similarly, a study by Naser et al. demonstrated that heart failure with preserved ejection fraction (HFpEF) was more common compared with heart failure with reduced ejection fraction (HFrEF) in patients diagnosed with Graves’ disease with a prevalence of 3.1 and 2.3%, respectively.^32^ The study also showed that high FT4 levels posed a significant risk for heart failure, with an increased risk of HFrEF when the FT4 level is higher than 4.2 ng/dL. In HFpEF, levels higher than 1.9 ng/dL did not show an additional effect on risk.^32^

This meta-analysis suggests that patients in the radioactive iodine group have the highest prevalence of heart failure contrary to the existing literature, indicating that antithyroid medication is associated with the highest risk of developing heart failure.^32^ Furthermore, studies have indicated that treatment with antithyroid medication for more than 24 months may enhance remission rate, but an extended duration of antithyroid medication poses an increased risk of developing heart failure.^15,42–44^ However, Okosieme et al. demonstrated that patients with unresolved hyperthyroidism after radioactive iodine ablation had a higher risk of developing major adverse cardiovascular events and all-cause mortality at 1 year compared with those treated with antithyroid medications.^29^ Similarly, the study by Metso et al. showed an increased risk of cardiovascular morbidity and mortality and hospitalization for cardiovascular complications, such as heart failure, atrial fibrillation, and hypertension, after radioactive iodine ablation.^27^ The variations in the risk of heart failure between the antithyroid medication and radioactive iodine ablation groups may be attributed to the fact that certain patients, experiencing persistent elevated thyroid hormones resulting from recurrent clinical or subclinical hyperthyroidism after treatment, face an increased risk of mortality and cardiovascular complications, irrespective of treatment modalities.^29,32^

Our study also demonstrated that the prevalence of heart failure is the lowest in patients who had thyroidectomy. Similarly, Ryödi et al. found that patients who underwent thyroidectomy had a lower risk of hospitalization due to cardiovascular events and the development of new-onset atrial fibrillation compared with the patients treated with radioactive iodine ablation.^28^ Patients undergoing total thyroidectomy exhibited an improvement in diastolic heart failure, as evidenced by the biomarker N-terminal pro-brain natriuretic peptide, in comparison with those treated with antithyroid medication.^45^ This is because surgical management can definitively eliminate the endogenous production of thyroid hormones, enabling a more rapid achievement of remission, which facilitates the resolution of cardiovascular complications and normalization of haemodynamics.^30^ Furthermore, a meta-analysis demonstrated a significant improvement in clinical outcomes for patients with Graves’ disease undergoing thyroidectomy, which may be attributed to advancements in surgical techniques and pre-surgery medication management, making it a more cost-effective option compared with other treatment modalities.^46^

*CI = confidence interval; df = degrees of freedom; IV = inverse variance; SE = standard error.*

*CI = confidence interval; df = degrees of freedom; IV = inverse variance; SE = standard error.*

This meta-analysis established that atrial fibrillation poses the highest risk of developing heart failure in patients with hyperthyroidism. Atrial fibrillation in hyperthyroidism may arise from a decrease in action potential, resulting in a reduced atrial refractory period and an increase in sympathetic tone, leading to a decrease in heart rate variability.^47^

Atrial fibrillation can lead to heart failure through various mechanisms, such as increased resting heart rate, increased heart rate response to exercise, shortened diastolic filling time due to irregular ventricular response and compromised effective atrial contractile function.^48^ Given that hyperthyroidism itself can contribute to heart failure, the presence of additional atrial fibrillation in patients may further elevate the risk of developing heart failure. Furthermore, similar to the study by Naser et al., our meta-analysis also showed that the presence of cardiovascular comorbidities, including anaemia, chronic kidney disease, hypertension, history of stroke or transient ischaemic attack, history of coronary artery disease and diabetes mellitus, had an increased risk of developing heart failure.^32^ The study also suggested that patients diagnosed with heart failure and no underlying cardiovascular risk factors have a better prognosis and outcome as their recovery is primarily determined by the duration required to attain a euthyroid state in contrast to patients with underlying cardiovascular diseases who are less likely to experience complete recovery.^32^

There were several limitations to this meta-analysis. Significant heterogeneity in the results was observed, possibly attributed to variations in the populations studied, differences in sample sizes and methodologies and incomplete reporting of results. Nonetheless, the utilization of random-effect models aimed to minimize the impact of heterogeneity on the outcomes. Additionally, the analysis of the funnel plot and Egger’s regression test suggested the presence of publication bias. Furthermore, the majority of studies did not report the diagnostic criteria for heart failure in the studied population. Moreover, with the exception of two studies, none categorized patients into HFpEF and HFrEF, thus precluding the possibility of performing a subgroup analysis.

## Conclusion

Heart failure occurs in 8% of patients diagnosed with hyperthyroidism, and the literature suggests that HFpEF is more prevalent in this population. The prevalence of heart failure is the lowest among patients who undergo thyroidectomy, implying that thyroidectomy may be effective for those who develop severe cardiovascular complications. The presence of cardiovascular risk factors and atrial fibrillation is associated with increased odds of developing heart failure in patients diagnosed with hyperthyroidism. Therefore, long-term follow-up and close monitoring are required for patients with overt hyperthyroidism to assess for congestive heart failure, as this condition is reversible upon appropriate treatment.

**Table 3:** Summary of risk factors for developing heart failure in patients diagnosed with heart failure

Risk factors	Odds ratio		LCI		UCI		p-value
Demographics	
Male	1.92	0.63	5.83	0.25
Smoking	1.47	0.65	3.32	0.35
Hyperthyroidism diagnosis				
Goitre	1.89	0.96	3.71	0.06
Graves’ disease	0.55	0.28	1.08	0.08
Cardiovascular risk factors				
Atrial fibrillation	17.09	4.86	60.17	<0.001*
Chronic kidney disease	6.40	3.72	11.01	<0.001*
Anaemia	5.04	3.17	8.00	<0.001*
Hypertension	4.84	2.72	8.62	<0.001*
History of stroke/transient ischaemic attack	4.28	1.72	10.65	0.002*
History of coronary artery disease	2.87	1.37	5.99	0.005*
Diabetes mellitus	2.36	1.41	3.96	0.001*
Chronic obstructive pulmonary disease	4.15	0.80	21.66	0.09
Dyslipidaemia	1.48	0.31	7.20	0.62

*Statistically significant at p<0.05.

*LCI = lower confidence interval; UCI = upper confidence interval.*
